# Intratumor Heterogeneity of ALK-Rearrangements and Homogeneity of EGFR-Mutations in Mixed Lung Adenocarcinoma

**DOI:** 10.1371/journal.pone.0139264

**Published:** 2015-09-30

**Authors:** Federica Zito Marino, Giuseppina Liguori, Gabriella Aquino, Elvira La Mantia, Silvano Bosari, Stefano Ferrero, Lorenzo Rosso, Gabriella Gaudioso, Nicla De Rosa, Marianna Scrima, Nicola Martucci, Antonello La Rocca, Nicola Normanno, Alessandro Morabito, Gaetano Rocco, Gerardo Botti, Renato Franco

**Affiliations:** 1 Pathology Unit, Istituto Nazionale Tumori “Fondazione G. Pascale”-IRCCS, Naples, Italy; 2 Department of Pathophysiology and Organ Transplantation, University of Milan Medical School, Milan, Italy; Division of Pathology, Fondazione IRCCS Ca’ Granda, Ospedale Maggiore Policlinico Milan, Italy; 3 Department of Biomedical, Surgical and Dental Sciences, University of Milan Medical School, Milan, Italy; Division of Pathology, Fondazione IRCCS Ca’ Granda, Ospedale Maggiore Policlinico Milan, Italy; 4 Thoracic Surgery and Lung Transplantation Unit, Fondazione IRCCS Ca’ Granda, Ospedale Maggiore Policlinico Milan, Italy; 5 Department of Oncology and Anatomic Pathology, Hospital of National Relevance (AORN) Vincenzo Monaldi, Naples, Italy; 6 Biogem scarl, Institute for Genetic Research “Gaetano Salvatore”, Ariano Irpino (Avellino), Italy; 7 Division of Thoracic Surgery, Department of Thoracic Surgical and Medical Oncology, Istituto Nazionale Tumori “Fondazione G. Pascale”-IRCCS, Naples, Italy; 8 Cell Biology and Biotherapy Unit, Istituto Nazionale Tumori “Fondazione G. Pascale”-IRCCS, Naples, Italy; 9 Medical Oncology Unit, Department of Thoracic Surgical and Medical Oncology, Istituto Nazionale Tumori “Fondazione G. Pascale”-IRCCS, Naples, Italy; Second University of Naples, ITALY

## Abstract

**Background:**

Non Small Cell Lung Cancer is a highly heterogeneous tumor. Histologic intratumor heterogeneity could be ‘major’, characterized by a single tumor showing two different histologic types, and ‘minor’, due to at least 2 different growth patterns in the same tumor. Therefore, a morphological heterogeneity could reflect an intratumor molecular heterogeneity. To date, few data are reported in literature about molecular features of the mixed adenocarcinoma. The aim of our study was to assess EGFR-mutations and ALK-rearrangements in different intratumor subtypes and/or growth patterns in a series of mixed adenocarcinomas and adenosquamous carcinomas.

**Methods:**

590 Non Small Cell Lung Carcinomas tumor samples were revised in order to select mixed adenocarcinomas with available tumor components. Finally, only 105 mixed adenocarcinomas and 17 adenosquamous carcinomas were included in the study for further analyses. Two TMAs were built selecting the different intratumor histotypes. ALK-rearrangements were detected through FISH and IHC, and EGFR-mutations were detected through IHC and confirmed by RT-PCR.

**Results:**

10/122 cases were ALK-rearranged and 7 from those 10 showing an intratumor heterogeneity of the rearrangements. 12/122 cases were EGFR-mutated, uniformly expressing the EGFR-mutated protein in all histologic components.

**Conclusion:**

Our data suggests that EGFR-mutations is generally homogeneously expressed. On the contrary, ALK-rearrangement showed an intratumor heterogeneity in both mixed adenocarcinomas and adenosquamous carcinomas. The intratumor heterogeneity of ALK-rearrangements could lead to a possible impact on the therapeutic responses and the disease outcomes.

## Introduction

Lung cancer represents a very heterogeneous tumor not only for its clinical and radiologic presentation but also for its histological and molecular features. Particularly, major histologic heterogeneity, characterized by a single tumor showing at least two different histologic types, and minor histologic heterogeneity with a single tumor showing just one histologic type but at least two different growth patterns, are generally recorded [1].

Adenocarcinoma (ADC) is the most frequently diagnosed histological type of primary lung cancer accounting for almost half of all lung cancers [2]. Histologic intratumoral heterogeneity in ADCs is expressed both in terms of a frequent minor heterogeneity due to the occurrence of several growth patterns, such as lepidic, acinar, papillary, micropapillary and solid adenocarcinoma in mixed adenocarcinomas (mADCs), and an unusual major heterogenity as in the adenosquamous lung carcinomas (AdSqLCs). According to the 2004 World Health Organization (WHO) classification, more than 80% of lung adenocarcinomas falls into the mixed subtype [3].

In recent years, major revisions have been made to the classification and grading of ADCs, leading to an improvement of the diagnosis of these tumors with a subsequent prognostic stratification. Thus, the 2011 IASLC/ATS/ERS (International Association for the Study of Lung Cancer/American Thoracic Society/European Respiratory Society) classification of lung adenocacinomas has introduced some significant revisions to the 2004 WHO ADCs classification [4]. In particular, a 5% increment in the comprehensive histologic subtyping of the different patterns of mADCs is recommended, in order to determine the predominant pattern. Many series have shown the clinicopathological relevance and the prognostic role of the 2011 IASLC/ATS/ERS classification being the predominant pattern of adenocarcinoma related to different prognosis [5]. Moreover, Sica et al. proposed a grading system based on the histologic subtyping of mADCs, providing a grading score based on the two main growth patterns [6]. This pattern-based scoring system has proved to improve both mADCs subclassification and better prognostic stratification [7].

In the past decade, the advancement on the treatment targeting mutated Epidermal Growth Factor Receptor (EGFR) and Anaplastic Lymphoma Kinase (ALK) have changed considerably the possibility of therapy for Non Small Cell Lung Cancer (NSCLC) patients. EGFR gene most common and clinically relevant mutations, accounting for approximately 80–90% of EGFR-mutated patients, occur in two hot spots in the tyrosine kinase (TK) domain: in-frame deletions in exon 19 (most frequently E746-A750) and L858R missense mutation in exon 21 leading to a base substitution of arginine to leucine at residue 858. The NSCLCs harboring these mutations are responsive to EGFR tyrosine inhibitors and kinase inhibitors (TKIs) [8]. Moreover, in AdSqLCs, a relatively high frequency of EGFR mutations (mut-EGFR) is described [9,10], thereby patients with AdSqLCs are treated as lung adenocarcinoma. Although the presence of two different histological components in AdSqLCs, identical mut-EGFR have been detected simultaneously in both components, suggesting that the adenocarcinoma and squamous cell carcinoma have a possible clonal origin [9].

ALK gene rearrangements (ALK-R) have been detected in approximately 5–7% of all NSCLCs, with enrichment in a selected NSCLCs according to the specific clinicopathological features, such as ADC histotype, never/former light smoker (<10 packs/year), young and EGFR/KRAS wild-type patients [11]. ALK-rearranged NSCLC patients are responsive to the treatment with specific inhibitors.

Several studies reported that mut-EGFR and ALK-R, generally mutually exclusive, are frequently associated with specific histological features within the framework of adenocarcinomas [11].

In literature, the main histologic subtypes ascribed to mut-EGFR are lepidic or mixed subtype with lepidic component [12,13] and papillary and micropapillary [13,14]. ALK-R are described in many ADC subtypes, including lepidic, papillary, micropapillary and acinar [15–17]. However, ALK-R are more frequently associated to the solid growth pattern with signet ring cell component and a mucinous cribriform pattern [16,18]. ALK-R are rarely described in AdSqLCs [19]. The high rate of morphological heterogeneity in ADCs/AdSqLCs could reflect a heterogeneous molecular profiling, suggesting differential predictive values with specific potential therapeutical approaches. Thus, the aim of the present study is to assess mut-EGFR, such as E746-A750 deletions (del E746-A750) and L858R mutation (mut L858R), and ALK-R in the different subtypes and/or growth patterns in a series of ADCs/AdSqLCs

## Materials and Methods

### Ethics Statement

Ethics Statement Patient accrual was conducted according to internal Review Board of the INT Fondazione Pascale (Naples, Italy) (CEI 556/10 of 12/3/2010), the Fondazione IRCCS “Ca’ Granda”—Ospedale Maggiore Policlinico (Milan, Italy) (179/2013 of 19/3/2013) and the AORN Vincenzo Monaldi(Naples, Italy). Written informed consent was obtained from all participants to the study. The INT Fondazione Pascale, the Fondazione IRCCS “Ca’ Granda”—Ospedale Maggiore Policlinico and the AORN Vincenzo Monaldi ethics committees specifically approved this study.

### Patient Cohort

A series of 590 NSCLCs tumors from patients undergoing surgical resection between 2006 and 2011 at the Istituto Nazionale Tumori “Fondazione G Pascale”-IRCCS, Naples, the Fondazione IRCCS “Ca’ Granda”—Ospedale Maggiore Policlinico, and the AORN Vincenzo Monaldi, Naples were collected. Among the 590 NSCLCs, 443 were ADCs, 19 AdSqLCs and 128 squamous cell carcinomas. Inclusion criteria for the present analysis were: i) a tumor size of at least 1 cm of the selected block, ii) a tumor showing at least 2 different histotypes or patterns of growth, iii) amount of available material in order to core the tissue cylinders with a 1 mm diameter for each different histotype or pattern in the sample. The following clinical and pathological parameters were evaluated for patients cohort: patient age at initial diagnosis, smoking habits, histologic grade, tumor stage, tumor recurrence or distant metastasis.

### Histologic Evaluation

Sections of 4 μm thickness from each block (with a mean of 3 blocks per tumor) were obtained and were stained with hematoxylin-eosin. All 590 cases were reviewed by two expert pulmonary pathologists (R.F. and E.L.M.) in order to identify the AdSqLCs and the mADCs subtypes.

105 mADCs and 17 AdSqLCs have been selected, according to a tumor size of at least 1 cm and the histologic criteria suggested by Cadioli et al [1],minor heterogeneity was recorded when the tumor showed just 1 histotype but at least 2 different patterns as in mADC, whereas major heterogeneity was considered present when a tumor presented at least 2 different histotypes as in AdSqLCs. All cases were reviewed according to the new IASLC/ATS/ERS classification that allows for five comprehensive histologic subtypes of lepidic, acinar, papillary, solid and micropapillary patterns by a percentage of 5% increments to determine a predominant pattern [4]. Finally,the grade of mADC has been redefined according to the pattern-based scoring system proposed by Sica et al. [6].

### TMAs building

Two tissue microarrays were built using 105 mADCs, 17 AdSqLCs and 8 non-neoplastic lung tissue samples. All areas representative of each different histological pattern have been selected on the H&E-stained slides for every single case. Tissue cylinders (1 mm diameter) as many as the different selected histologic patterns were punched from each ‘donor’ tissue block and brought into one recipient paraffin block (3×2.5 cm) using a semiautomated tissue arrayer (Galileo TMA).

### Immunohistochemistry Analysis for EGFR mutations L858R and deletion E746-A750

Immunohistochemical staining was carried out on TMAs slides using EGFR mutation-specific antibodies in order to identify L858R point mutation in exon 21 and E746_A750 deletion in exon 19.

Paraffin slides was deparaffinized in xylene and rehydrated through graded alcohols. Antigen retrieval was performed with slides heated in EDTA buffer (pH 9.0) in a bath for 20 min at 97°C. After antigen retrieval, the slides were allowed to cool. The slides were rinsed with TBS and the endogenous peroxidase was inactivated with 3% hydrogen peroxide. After protein block (BSA 5% in PBS 1×), the slides were incubated with monoclonal rabbit antibodies against L858R mutation (dilution 1:100, clone 43B2 Cell Signaling Technology) and E746_A750 deletion (dilution 1:100, clone 6B6 Cell Signaling Technology) at 4°C overnight. The sections were incubated with biotinylated anti-rabbit antibody for 40 minutes at room temperature. Immunoreactivity was visualized by means of avidin-biotin-peroxidase complex kit reagents (Novocastra, Newcastle, UK) as the chromogenic substrate. Finally, sections were weakly counterstained with haematoxylin and mounted. As described in previous studies [20], the IHC scoring was based on the cytoplasmic and/or membrane-staining intensity, as follows: no staining, score 0; weak staining in ≥10% of tumor cells, score 1+; moderate staining in ≥10% of tumor cells, score 2+; strong staining in ≥10% of tumor cells, score 3+. Furthermore, all cases with IHC score 1+,2+,3+ were analyzed through molecular analysis to confirm mut-EGFR.

### Real-time PCR for EGFR mutations L858R and deletion E746-A750

A real-time polymerase chain reaction (PCR)-based assay that uses mutant-specific probes (EntroGen Inc USA), to identify the presence of EGFR mutations in exons 18,19,20 and 21.

Genomic DNA was extracted according to manufacturer’s protocol.

### Fluorescence in situ hybridization for ALK gene rearrangements

Fluorescence in situ hybridization (FISH) analysis was performed on unstained 5 μm thick TMAs slides, to detect ALK-R. Deparaffinization of sections was carried out with two 10 min immersions in bioclear, followed by three 3 min immersions in ethanol 100, 70 and 50%. The slides were rinsed in distilled water and immersed in Vysis pretreatment solution (1 M sodium thiocyanate) at 80°C for 10 min, and in protease solution (previously warmed to 37°C) for 10 min, washed with purified water, air-dried, and dehydrated in ascending grades of alcohol. The used probe was the commercially available ALK probe (Vysis LSI ALK Dual Color, Break Apart Rearrangement Probe; Abbott Molecular, Abbott Park, IL) consisting of two fluorescent probes, one red and one green signal, flanking the ALK break point. Denaturation and hybridization of the tissue sections were performed using the Thermobrite system (Abbott Molecular Inc. Des Plaines, IL): 75°C for 5 min for the denaturation process and 37°C for 17 hours for the hybridization of the probes. The slides were then washed with 0.4X saline- sodium citrate (SSC) solution at 70°C for 2 min and 2X SSC at room temperature for 3–5 min. Lastly, 10 μL of DAPI was applied on the slides. The fluorescence signals were evaluated under epifluorescence microscope (Olympus) and the image acquisition was done by CCD microscopy camera. FISH for ALK locus rearrangements was considered positive in relation to two different patterns: (1) the classic break-apart pattern with one fusion signal and two separated orange and green signals (a distance ≥2 signal diameters between two signals); (2) the atypical pattern with a single red signal without a corresponding green signal in addition to fused signals.[21] A sample was considered positive if >15% of the cells counted out of minimum 60 in at least one tissue core were positive.

### Immunohistochemistry Analysis for ALK protein expression

Immunohistochemical staining was performed on 4 μm thick TMAs sections. Immunohistochemistry (IHC) analysis was performer using the VENTANA anti-ALK (D5F3) Rabbit Monoclonal Primary Antibody, used with the OptiView DAB IHC Detection Kit and OptiView Amplification Kit (Ventana Medical Systems USA), as a fully automated assay on the Ventana BenchMark XT processor. A binary scoring system (positive or negative for ALK status) was adopted to evaluate the staining results, according to the manufacture’s scoring algorithm. The samples were considered positive when tumor cells (any percentage of positive tumor cells) showed a strong granular cytoplasmic staining.

### Statistical Analysis

Pearson Χ-square test was performed to determine the association of clinical characteristics with status of protein expression. Spearman rank test was used to assess the correlation between protein phenotypes. The logrank test was used to compare survival distributions between positive and negative staining groups. P< 0.05 (2-sided) was considered to be statistically significant. Data analysis and summarization were conducted using SPSS 20.0 for Mac (SPSS Inc., Chicago, Ill).

## Results

### Clinical and Pathological Characteristics of patients

**Fig 1:**
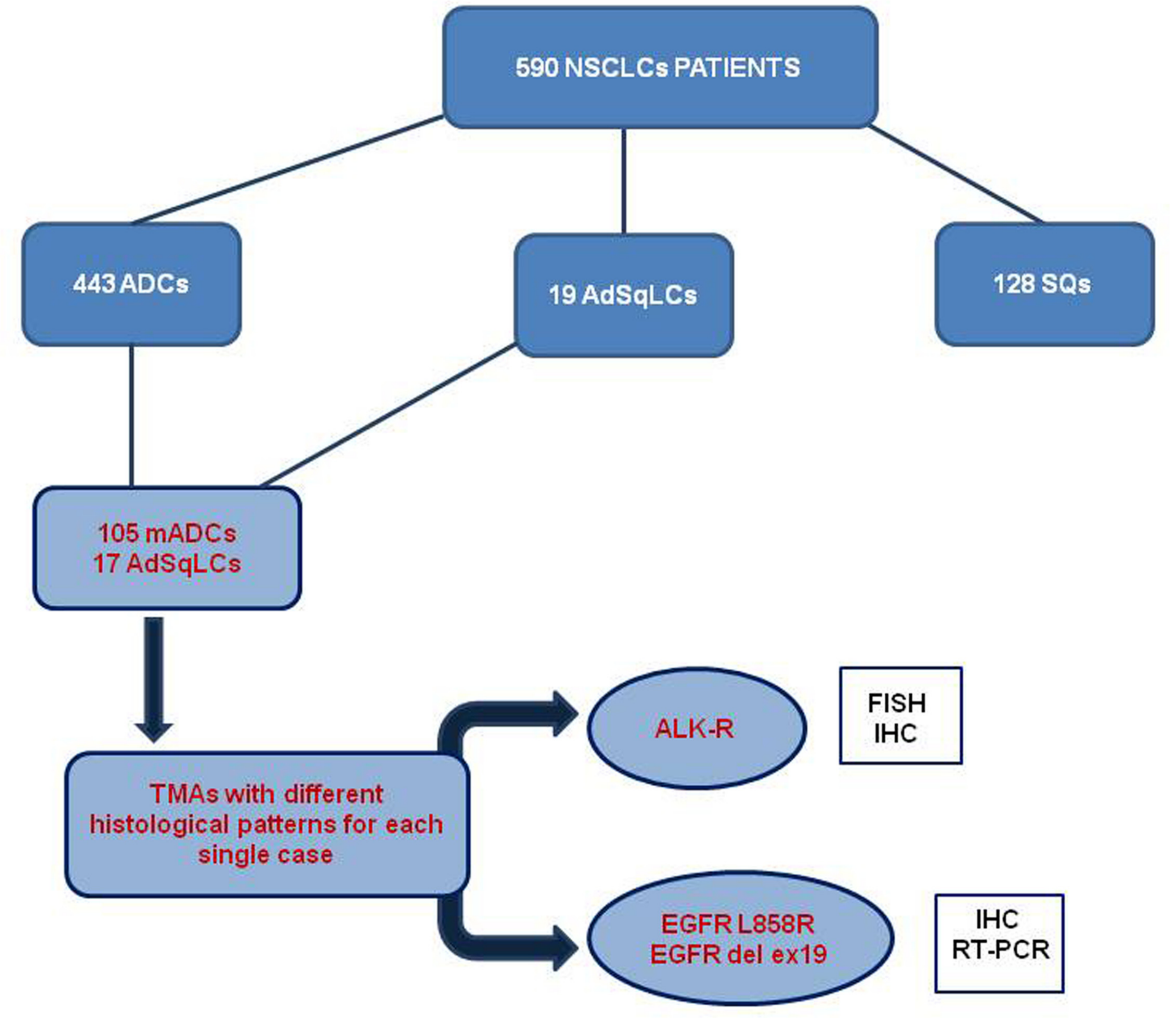
Study Profile. NSCLC: Non Small Cell Lung Cancer; ADC:Adenocarcinoma; AdSqLC: Adenosquamous Lung carcinoma; SQ: Squamous Cell carcinoma; mADCs: mixed adenocarcinomas; ALK-R: ALK-rearrangments.

Among the 590 NSCLCs revised, 105 mADCs and 17 AdSqLCs were included in the present analysis (Fig 1). Clinical and pathological features of 122 patients are shown in Table 1. 85 out of 122 (69.7%) were male patients. The mean age was 64 years (range 44–82). 64 out of 87 were current smokers. Among all patients, 53.2% were stage I, 24.6% stage II, 20.5% stage III, and 1.6% stage IV. Of the 17 AdSqLCs, 10 were grade II, 6 grade III and 1 grade I. Using the grading scheme proposed by Sica et al [6], of the 105 mADCs, 63 were grade III, 31 grade II and 11 grade I. The majority of the mADCs were composed of two histological patterns of growth (71 cases, 67.6%), 26 cases (24.7%) of three patterns, 6 cases (5.7%) of four patterns and only 2 samples (1.9%) of more than four patterns. In most mADCs the predominant pattern was the solid pattern (51cases, 48.6%), in 38 cases (36.2%) was the acinar pattern, followed by the lepidic pattern (10 cases; 9.5%), papillary pattern (5 cases; 4.7%) and only 1 case with micropapillary pattern.

**Table 1:** Clinical and pathological features of patients.

Characteristics	No. of cases(%)	mADCs	AdSqLCs
**All cases**	122	105	17
**Age, years**			
>64 y	64(52.4%)	56(53.3%)	8(47.1%)
<64 y	58(47.5%)	49 (46.6%)	9(52.9%)
**Gender**			
male	85(69.7%)	74 (70.4%)	11(64.7%)
female	37(30.3%)	31(29.5%)	6(35.3%)
**Smoking Status**			
Smoker	64(52.4%)	56(53.3%)	8(47.1%)
Never and/or Light Smoker	16(13.1%)	15(14.3%)	1(5.8%)
Ex-smoker	7(5.7%)	3(2.8%)	4(23.5%)
NA*	35(28.7%)	31(29.5%)	4(23.5%)
**Disease stage**			
IA	33(27%)	31(29.5%)	2(11.8%)
IB	32(26.2%)	27(25.7%)	5(29.4%)
IIA	20(16.4%)	17(16.2%)	3(17.6%)
IIB	10(8.2%)	9(8.6%)	1(5.9%)
IIIA	25(20.5%)	19(18.1%)	6(35.3%)
IV	2(1.6%)	2(1.9%)	-
**Grade**		**Sica score**	
I	12(9.8%)	11(10.5%)	1(5.8%)
II	41(33.6%)	31(29.5%)	10(58.8%)
III	69(56.5%)	63(60%)	6(35.3%)
**ALK Status**			
ALK wt	112(91.8%)	98(93.3%)	14(88.2%)
ALK-R	10(8.2%)	7(6.6%)	3(17.6%)
**EGFR Status**			
del ex 19	10(8.2%)	10(9.5%)	-
L858R ex 21	2(1.6%)	2(1.9%)	-
wt	110(90.2%)	93(88.6%)	17(100%)
**Histological patterns**			
Acinar		81(77.1%)	
Solid		73(69.5%)	
Papillary		40(38.0%)	
Lepidic		27(25.7%)	
Mucinous		16(15.2%)	
Signet-ring cell		9(8.5%)	
Micropapillary		7(6.6%)	
**Predominant Pattern**			
Solid		51(48.6%)	
Acinar		38(36.2%)	
Lepidic		10(9.5%)	
Papillary		5(4.7%)	
Micropapillary		1(0.95%)	

NA: not available; Wt: wild type, mADCs: mixed adenocarcinomas, AdSqLCs: adenosquamous lung carcinoma,ALK: Anaplastic Lymphoma Kinase, EGFR: Epidermal Growth Factor Receptor.

### EGFR Results

#### EGFR IHC and RT-PCR

12/122 cases were EGFR-mutated: specifically, 10 cases carrying del E746-A750 and 2 mut L858R. 10 cases positive by immunohistochemistry (IHC) were confirmed by Real time PCR, but no material was available for the other two cases. Clinical and pathological features of the EGFR-mutated patients are shown in Table 2. 7 out of 12 patients were females patients. The mean age of EGFR-mutated patients was 63.4 (range 49–78). 5 cases out of 12 were ex-smoker/nonsmokers, 3 current smokers and no smoking habits were available for 4 patients. Of the 12 EGFR-mutated cases, 7 were of 12 grade II and 5 of grade III. Most EGFR positive cases were recorded in stage I (5/12) and stage II (5/12). The statistical analysis showed no correlation between mut-EGFR and clinicopathological features of patients.

**Table 2:** Clinical and pathological features of patients harboring mut-EGFRand ALK-R.

Characteristics	No, of cases mut-EGFR	No, of cases ALK-R	No, of cases ALK-R and mut- EGFR
**All cases**	**12**	**10**	**2**
**Age, years**			
>64 y	6(50%)	7(70%)	2(10%)
<64 y	6(50%)	3(30%)	0
**Gender**			
male	5(41.6%)	10(100%)	2(10%)
female	7(58.3%)	0	0
**Smoking Status**			
Smoker	3(25%)	2(20%)	0
Never and/or Light Smoker	3(25%)	2(20%)	0
Ex-smoker	2(16.6%)	3(30%)	1(5%)
NA*	4(33.3%)	3(30%)	1(5%)
**Disease stage**			
IA	3(25%)	3(30%)	0
IB	2(16.6%)	3(30%)	1(5%)
IIA	4(33.3%)	2(20%)	0
IIB	1(8.3%)	1(10%)	1(5%)
IIIA	2(16.6%)	1(10%)	0
IV	0	0	0
**Grade**			
I	0	0	0
II	7(58.3%)	3(30%)	1(5%)
III	5(41.6%)	7(70%)	1(5%)
**Subtype**			
mADCs	12(100%)	7(70%)	2(10%)
AdSqLC	0	3(30%)	0
**No. of cases with heterogeneity**	1(8.3%)	7(70%)	0 mut EGFR, 2 ALK-R,
**No. of patterns with aberration**			
Acinar	11	4	2 mut EGFR, 0 ALK-R
Solid	7	9	2 mut EGFR, 2 ALK-R
Papillary	5	1	1 mut EGFR, 0 ALK-R
Lepidic	4	1	0 mut EGFR, 0 ALK-R
Mucinous	2	0	1 mut EGFR, 0 ALK-R
Signet-ring cell	2	1	1 mut EGFR, 1 ALK-R
Micropapillary	1	0	1 mut EGFR, 0 ALK-R

NA: not available; Wt: wild type, mADCs: mixed adenocarcinomas, AdSqLCs: adenosquamous lung carcinoma,ALK: Anaplastic Lymphoma Kinase, EGFR: Epidermal Growth Factor Receptor.

#### Intratumoral homogeneity of EGFR mutations

All 12 EGFR-mutated cases were mADCs and 11 out of 12 cases uniformly expressed the mutated receptor in all histologic patterns, except case n. 8. The details are summarized in Table 3.

**Table 3:** Clinical and Histological features of ALK-rearranged and EGFR-mutated patients.

Case	Sex	Age	Stage	Smoker	Grade	Sica Score	Patterns	ALK FISH (*^1^)	ALK IHC	EGFR L858R	EGFR Del	EGFR RT-PCR
**1**	**M**	**49**	**IIIA**	**YES**	**III**	**5(3,2)**	**solid**	**NR**	**-**	**-**	**40%**	**del**
							**solid-signet**	**NR**	**-**	**-**	**50%**	**del**
							**acinar**	**NR**	**-**	**-**	**40%**	**del**
**2**	**F**	**63**	**IIA**	**NO**	**II**	**4(1,3)**	**lepidic**	**NR**	**-**	**-**	**40%**	**del**
							**solid**	**NR**	**-**	**-**	**50%**	**del**
							**acinar**	**NR**	**-**	**-**	**40%**	**del**
**3**	**F**	**52**	**IA**	NA*	**II**	**4 (2,2)**	**acinar**	**NR**	**-**	**30%**	**-**	**mut L858R**
							**papillary**	**NR**	**-**	**40%**	**-**	**mut L858R**
							**lepidic**	**NR**	**-**	**30%**	**-**	**mut L858R**
**4**	**M**	**78**	**IB**	**EX**	**III**	**5 (3,2)**	**solid**	**R(30%)**	**+**	**-**	**30%**	**del**
							**solid-signet**	**a R(20%)**	**+**	**-**	**40%**	**del**
							**micropapillary**	**NR(<5%)**	**-**	**-**	**30%**	**del**
							**papillary**	**NR(<5%)**	**-**	**-**	**30%**	**del**
							**acinar**	**NR(<5%)**	**-**	**-**	**60%**	**del**
**5**	**M**	**75**	**IB**	**EX**	**III**	**AdSqLC**	**solid-signet**	**NR(<5%)**	**-**	**-**	**-**	**/**
							**solid**	**R(30%)**	**+**	**-**	**-**	**/**
							**acinar**	**NR(<5%)**	**-**	**-**	**-**	**/**
							**squamous**	**NR(<5%)**	**-**	**-**	**-**	**/**
**6**	**M**	**65**	**IB**	**EX**	**III**	**AdSqLC**	**acinar**	**NR(<5%)**	**-**	**-**	**-**	**/**
							**solid**	**R(20%)**	**+**	**-**	**-**	**/**
							**squamous**	**NR(<5%)**	**-**	**-**	**-**	**/**
**7**	**F**	**52**	**IA**	**EX**	**II**	**3 (2,1)**	**papillary**	**NR**	**-**	**-**	**70%**	**del**
							**lepidic**	**NR**	**-**	**-**	**70%**	**del**
**8**	**F**	**66**	**IIA**	**NO**	**II**	**4(2,2)**	**papillary**	**NR**	**-**	**-**	-	**del**
							**mucinous**	**NR**	**-**	**-**	**40%**	**del**
							**acinar**	**NR**	**-**	**-**	**50%**	**del**
**9**	**M**	**67**	**IA**	NA*	**III**	**5 (3,2)**	**solid**	**R(20%)**	**+**	**-**	**-**	**/**
							**acinar**	**R(20%)**	**+**	**-**	**-**	**/**
							**lepidic**	**R(20%)**	**+**	**-**	**-**	**/**
**10**	**M**	**77**	**IA**	NA*	**III**	**5 (3,2)**	**solid**	**R(30%)**	**+**	**-**	**-**	**/**
							**acinar**	**R(20%)**	**+**	**-**	**-**	**/**
**11**	**M**	**77**	**IIB**	NA*	**II**	**5 (2,3)**	**acinar**	**NR(<5%)**	**-**	**-**	**30%**	**del**
							**mucinous**	**NR(<5%)**	**-**	**-**	**10%**	**del**
							**solid**	**R(30%)**	**-**	**-**	**20%**	**del**
**12**	**M**	**68**	**IA**	NA*	**II**	**3 (2,1)**	**acinar**	**NR**	**-**	**40%**	**-**	**mut L858R**
							**lepidic**	**NR**	**-**	**30%**	**-**	**mut L858R**
**13**	**M**	**44**	**IIA**	**NO**	**II**	**5 (2,3)**	**acinar**	**R(30%)**	NA*	**-**	**-**	**/**
							**solid**	**R(20%)**	NA*	**-**	**-**	**/**
							**papillary**	**NR(<5%)**	NA*	**-**	**-**	**/**
**14**	**M**	**60**	**IA**	**YES**	**III**	**AdSqLC**	**solid**	**R(20%)**	**+**	**-**	**-**	**/**
							**squamous**	**NR(<5%)**	**-**	**-**	**-**	**/**
**15**	**M**	**47**	**IIA**	**NO**	**II**	**4 (2,2)**	**papillary**	**a R(20%)**	**+**	**-**	**-**	**/**
							**acinar**	**a R(20%)**	**+**	**-**	**-**	**/**
**16**	**M**	**66**	**III**	**YES**	**III**	**5 (3,2)**	**solid**	**R(30%)**	**+**	**-**	**-**	**/**
							**acinar**	**NR(<5%)**	**-**	**-**	**-**	**/**
**17**	**F**	**66**	**IIA**	NA*	**III**	**5 (3,2)**	**solid**	**NR**	**-**	**-**	**30%**	NA*
							**acinar**	**NR**	**-**	**-**	**10%**	NA*
**18**	**F**	**61**	**IIA**	**NO**	**II**	**4 (2,2)**	**acinar**	**NR**	**-**	**-**	**30%**	NA*
							**papillary**	**NR**	**-**	**-**	**10%**	NA*
**19**	**F**	**66**	**IB**	**YES**	**III**	**5 (3,2)**	**solid**	**NR**	**-**	**-**	**40%**	**del**
							**acinar**	**NR**	**-**	**-**	**10%**	**del**
**20**	**M**	**63**	**IIIA**	**YES**	**III**	**5 (3,2)**	**acinar**	**NR**	**-**	**-**	**40%**	**del**
							**solid**	**NR**	**-**	**-**	**30%**	**del**
							**papillary**	**NR**	**-**	**-**	**40%**	**del**

NA*: not available; ALK: Anaplastic Lymphoma Kinase, EGFR: Epidermal Growth Factor Receptor, R: ALK-rearranged, a R: ALK-rearranged with atypical FISH pattern.

(* ^**1**^ **): % of neoplastic cells with ALK-R.**

### ALK Results

#### ALK FISH and Immunohistochemistry

10 out of 122 (8.2%) cases were ALK-rearranged cases identified through FISH. Clinical and pathological features of the ALK-rearranged patients are shown in Table 2. All 10 patients were males with a mean age of 65.6 years (range 44–78). 5 cases out of 7 were ex-smokers/nonsmokers, 2 current smokers. Most ALK-rearranged cases were grade III (7/10) and stage I (6/10). 2 out of 10 cases (samples 4 and 15) ALK-rearranged showed the atypical FISH pattern. 9 out of 10 ALK-rearranged by FISH cases are analyzed through IHC. Thus, 8 out of 9 ALK-rearranged through FISH cases were also positive by IHC, while only 1 is ALK IHC negative. The statistical analysis showed a significant relation between ALK-R and male gender (P *value* = 0.029). However, no statistical correlation between ALK-R and another clinicopathological features of patients was present.

#### Intratumor heterogeneity of ALK-R

7/10 ALK-rearranged cases were mADCs and 3/10 were AdSqLCs. Particularly, 7 out of 10 showed an intratumor heterogeneity relative to ALK-R presence. In all cases, ALK negative cores showed a percentage of ALK-R in neoplastic cell <5%. Thus, ALK-R in all 3 AdSqLCs were exclusively found in the adenocarcinoma component and, in mADC, the solid pattern showed ALK-R more frequently than the other patterns of growth, since 9 out of 10 ALK rearranged cases include solid growth pattern. The details of the histologic patterns are summarized in Table 3. The solid pattern showed ALK-R more frequently (9 out of 10 cases) than the others patterns of growth.

### Patients with Concomitant ALK-R and del-ex19 EGFR

**Fig 2:**
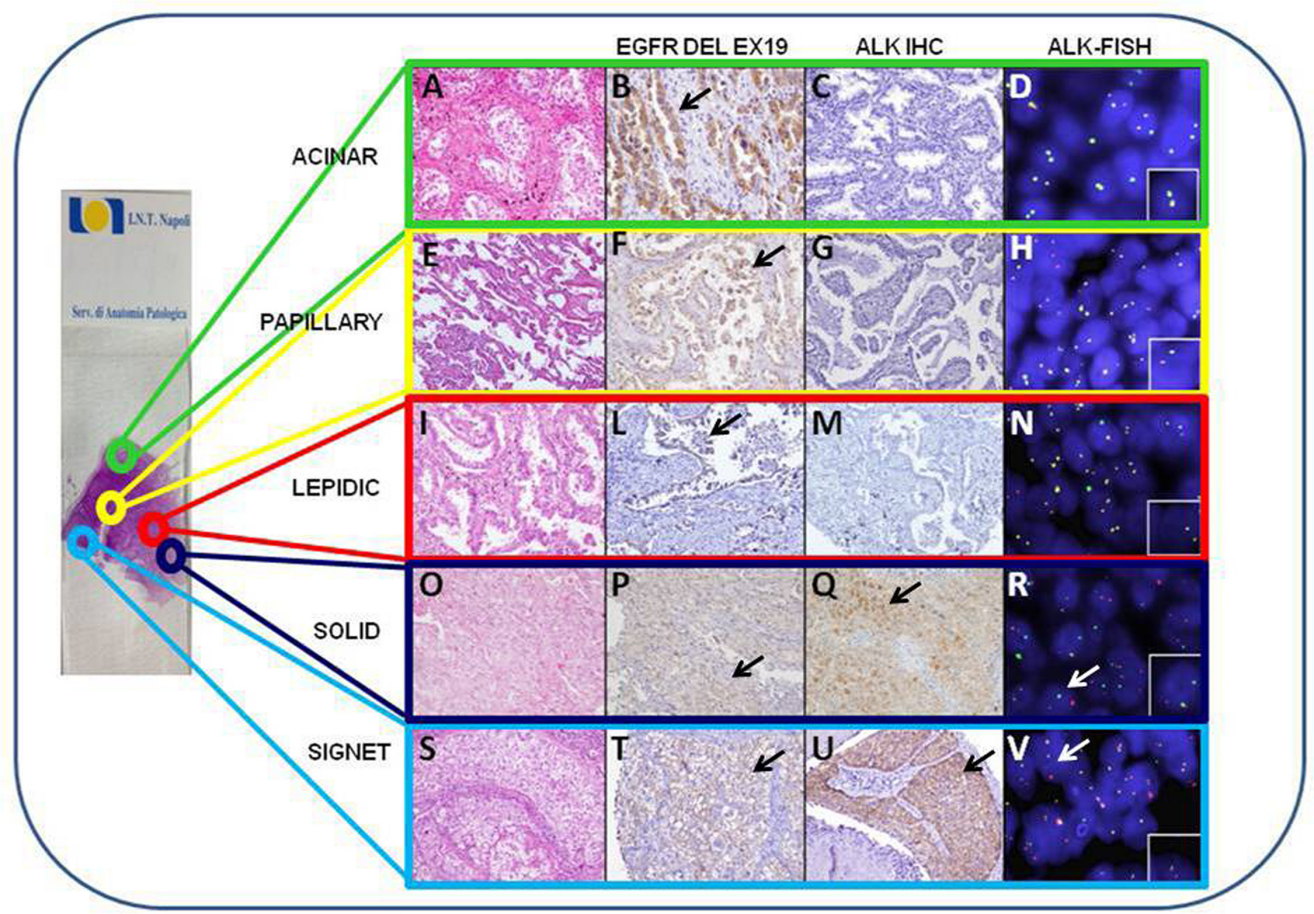
Representative results of the different areas of a mADC with coexistence of del E746-A750 EGFR and ALK-R (case 4). Tumoral area of standard H&E slide with microphotographs of each single growth pattern is identified by different colored arrows/box. Acinar pattern: A, B, C, D. Papillary pattern: E, F, G, H. Lepidic pattern: I, L, M, N. Solid pattern: O, P, Q, R. Solid-signet ring cell pattern: S, T, U, V. In the first column: Hematoxylin and Eosin staining for each histological pattern (20X). In the second column: del E746-A750 EGFR immunopositivity in all patterns. (20X) The black arrows indicate the positive staining: acinar (B), papillary (F), lepidic (L), solid (P), signet (T). In the third column: ALK IHC results, acinar (C), papillary (G) and lepidic (M) patterns are negative; solid(Q) and solid-signet ring cell (U) patterns are positive, indicated by the black arrows. In the fourth column: ALK FISH results, acinar (D), papillary(H) and lepidic(N) are negative; solid pattern (R) shows the classic break-apart pattern with one fusion signal and two separated orange and green signals, indicated by the white arrows. Solid-signet ring cell pattern (V) shows the atypical FISH pattern with fused signals and one single red signal without a corresponding green signal, indicated by the white arrows.

In our series, two males patients (samples 4 and 11, tab.2) had concomitant ALK-R and del E746-A750. Both cases showed an intratumor heterogeneity of ALK-R, while a homogeneous expression of del E746-A750 in all patterns was found. Clinical and pathological features are shown in Table 3. The histological features of case 4 are shown in Fig 2.

## Discussion

The intratumor heterogeneity in most human cancers is considered a great limitation for the correct diagnosis, the prognostic stratification and a successful treatment of the disease. The intratumor heterogeneity is the result of both the genetic disorders and the influence of the tumor microenvironment, both potentially reflecting on a variability of morphological features [22]. Thus, the balance of different tumor cells populations within the tumor should significantly determine the tumor progression and the cell survival under adverse conditions of the dynamic microenvironment and tumor resistance to specific treatment [23,24].

Recently, a multidisciplinary platform have radically systematized the characterization of lung ADC on histological, radiological, clinical and molecular levels. Particularly, the predominant pattern of tumoral growth in mADC and molecular features driving biological treatment have been greatly emphasized [4].

Although molecular characterization is crucial for the choice of specific therapeutic treatment, according to our knowledge, only few studies have analyzed mut-EGFR and ALK-R in series of mADC and/or AdSqLCs.

It has been proposed that lung adenocarcinoma development occurs from atypical adenomatous hyperplasia (AAH) through bronchioloalveolar (BAC), redefined as lepidic, and finally invasive adenocarcinoma, being the solid pattern the less differentiated adenocarcinoma component [25]. Furthermore, it has been reported that the solid subtype in invasive adenocarcinomas is an independent poor prognostic factor and negative predictor for adjuvant chemotherapy. In addition, although mut-EGFR is not frequent in solid predominant lung adenocarcinoma, EGFR-mutated adenocarcinomas with solid subtype have poor response to EGFR TKIs [26].

Previous studies have demonstrated EGFR abnormalities, as mutations and gene copy gain, in the progression of lung adenocarcinoma. In fact, multifocal lung adenocarcinomas with AAH and BAC (lepidic) patterns develop at 3 to 5 weeks of age in transgenic mice expressing the del E748-A752 mutant version of mouse EGFR driven by the surfactant protein C promoter [27]. In addition, it has variously demonstrated that those mutations are early detected in lung cancer development, such as in preinvasive lesions, whereas copy number gains are later events associated with the invasive phenotype and advanced stages of disease [28,29]. Thus, in the microdissected BAC (lepidic) pattern, EGFR mutations were commonly found [30]. Our data confirms these previous reports, as 11 out of 12 EGFR-mutated cases show a homogeneous EGFR mutation pattern in all histological patterns. The homogeneous distribution of EGFR mutations in all adenocarcinoma patterns of growth supports the hypothesis that they are an early genetic event, often associated to lepidic component.

In our series, mut-EGFR are more frequent in ADCs with acinar growth pattern (11 out of 12 EGFR-mutated cases). The response to EGFR-TKIs in homogeneous tumors is expected to be good, whereas it should be unsatisfactory in heterogeneous tumors. In fact, Chen et al compared 180 pairs of lung adenocarcinoma samples: primitive and node metastasis, primitive and distant metastasis, multiple lung nodules and metachrounous lung adenocarcinomas. The study concluded that EGFR mutational pattern heterogeneity frequency is relatively low, more commonly observed in multiple lung tumors, and that 38,2% of heterogenous pattern patients showed a variable tumor response to EGFR TKIs, with frequent development of EGFR resistance [31]. In addition, it has been reported that the intratumor heterogeneity of mut-EGFR is extremely rare, probably due to the use of less sensitive mut-EGFR detection assays [32,33]. In our study, we confirm by using highly sensitive RT-PCR the presence of mut-EGFR, in the cases identified through an immunohistochemical approach. Moreover, immunohistochemical homogeneous expression of mut-EGFR suggests that they are generally driver mutations, being present all over the tumor without clonal diversification. On the other hand, in our series, ALK-R showed a different distribution in the several growth patterns of mADCs. Previous studies have stated a relationship between ALK-R and the adenocarcinoma histotype, particularly in ADC including solid growth pattern and solid signet-ring variant. The results of the present study show an intratumor heterogeneity of ALK-R both in mADCs and AdSqLCs. In fact, 7 out of 10 cases harboring ALK-R have shown a diversified intratumoral distribution. Particularly, ALK-R are generally observed in solid patterns of mADCs and exclusively in the adenocarcinoma areas of AdSqLCs. Furthermore, 9 out of 10 ALK-rearranged sample cases showed the rearrangements in the solid component regardless of the other subtypes within the tumor. Moreover, solid pattern being frequently included in high Sica grading, ALK-R is significantly related to high grade tumors, as previously reported [11]. In our series, heterogeneous presence of ALK-R could suggest that the acquisition of this aberration is late during cancer progression.

In this view, a possible different frequency of mut-EGFR and ALK-R related to the different histologic subtype could suggest a different behavior of these driver mutations in ADC, particularly in lepidic and solid growth patterns.

In addition, in our series two cases were characterized by the concomitant presence of del E746-A750 and ALK-R. The distribution of del E746-A750 were not restricted to specific patterns, in contrast to ALK-R. Thus, in both cases, del E746-A750 EGFR was observed in all patterns and ALK-R only in solid and signet ring cell component. Previous studies have reported cases of concomitant EGFR mutation and ALK-R even with a low frequency [34–37]. In our series, the cases carrying both alterations represent the 17% of EGFR-mutated and the 20% of ALK-rearranged, underlying that in the context of mixed tumors the frequency of concomitant mutations could be higher. Thus, regarding patients harboring both mutations, it could open the possibility of different target therapeutic strategies.

In conclusion, our data show that the expression of ALK-R is heterogeneous compared to mut-EGFR in mADC and AdSqLCs. The results of this study raise several important issues regarding the intratumor heterogeneity of ALK-R such as the clinical and therapeutic implications, since different response to ALK inhibitors could be due to genetic heterogeneity, possibly strictly related to ADC “morphologic mosaic”. Further studies are required in order to clarify the causes and the significance of ALK-R intratumor heterogeneity.
