# Correction: Intratumor Heterogeneity of ALK-Rearrangements and Homogeneity of EGFR-Mutations in Mixed Lung Adenocarcinoma

**DOI:** 10.1371/journal.pone.0141521

**Published:** 2015-10-21

**Authors:** Federica Zito Marino, Giuseppina Liguori, Gabriella Aquino, Elvira La Mantia, Silvano Bosari, Stefano Ferrero, Lorenzo Rosso, Gabriella Gaudioso, Nicla De Rosa, Marianna Scrima, Nicola Martucci, Antonello La Rocca, Nicola Normanno, Alessandro Morabito, Gaetano Rocco, Gerardo Botti, Renato Franco

There is an error in the citation. The first author’s surname is: Zito Marino. The correct citation is: Zito Marino F, Liguori G, Aquino G, La Mantia E, Bosari S, Ferrero S, et al. (2015) Intratumor Heterogeneity of ALK-Rearrangements and Homogeneity of EGFR-Mutations in Mixed Lung Adenocarcinoma. PLoS ONE 10(9): e0139264. doi:10.1371/journal.pone.0139264
